# Exploring the influencing factors of patient safety competency of clinical nurses: a cross-sectional study based on latent profile analysis

**DOI:** 10.1186/s12912-024-01817-z

**Published:** 2024-03-04

**Authors:** Chunling Tai, Dong Chen, Yuhuan Zhang, Yan Teng, Xinyu Li, Chongyi Ma

**Affiliations:** 1 Nursing Department, The Second Affiliated Hospital Zhejiang University School of Medicine, No.88 Jiefang Road, Hangzhou, 310009 Zhejiang Province China; 2 Nursing Department, Heilongjiang Nursing College, Harbin, 150086 Heilongjiang Province China; 3 Student Affairs Office, The Second Affiliated Hospital of Harbin Medical University, Harbin, 150086 Heilongjiang Province China; 4 Department of Ophthalmology, The Second Affiliated Hospital of Harbin Medical University, Harbin, 150086 Heilongjiang Province China; 5 Department of Cardiology, The Second Affiliated Hospital of Harbin Medical University, No.256 Xuefu Road, Harbin, 150086 Heilongjiang Province China

**Keywords:** Clinical nurses, Patient safety competency, Latent profile analysis, Error management climate, Psychological security, Proactive behavior

## Abstract

**Background:**

Clinical nurses play an important role in ensuring patient safety. Nurses’ work experience, organizational environment, psychological cognition, and behavior can all lead to patient safety issues. Improving nurses’ attention to patient safety issues and enhancing their competence in dealing with complex medical safety issues can help avoid preventable nursing adverse events. Therefore, it is necessary to actively identify the latent profiles of patient safety competency of clinical nurses and to explore the influencing factors.

**Methods:**

A cross-sectional design was conducted. A total of 782 Chinese registered nurses were included in the study. Demographic characteristics questionnaire, Error Management Climate scale, Security Questionnaire, Proactive Behavior Performance scale and Patient Safety Competency Self-Rating Scale of Nurses were used. Latent profile analysis (LPA) was performed to categorize nurses into latent subgroups with patient safety competency differences. Multinomial logistic regression was conducted to explore the influencing factors of nurses’ patient safety competency (PSC) in different latent profiles.

**Results:**

A total of 782 questionnaires were valid. Nurses’ PSC was positively related to error management climate, and psychological safety and proactive behavior. The PSC score was 121.31 (SD = 19.51), showing that the PSC of clinical nurses was at the level of the medium on the high side. The error management climate score was 70.28 (SD = 11.93), which was at a relatively high level. The psychological safety score was 61.21 (SD = 13.44), indicating a moderate to low level. The proactive behavior score was 37.60 (SD = 7.33), which was at a high level. The latent profile analysis result showed that three groups of profile models were fitted acceding to the evaluation of PSC. They were defined as Low-competency Group (74 (9.5%)), Medium-competency Group (378 (48.3%)) and High-competency Group (330 (42.2%). Working years, professional titles, departments, error management climate, psychological security and proactive behavior were the influencing factors of PSC in three latent profiles.

**Conclusions:**

The PSC of clinical nurses had obvious classification characteristics, and the main influencing factors were working years, professional titles, working departments, error management climate, psychological security and proactive behavior. This study suggests that managers should pay attention to the continuous cultivation of patient safety competence among clinical nurses, provide targeted intervention measures for nurses at different work stages, professional titles, and departments, and use efficient management strategies to create a positive error management atmosphere. In patient safety management, providing nurses with more psychological security is conducive to stimulating more proactive behaviors and continuously improving the level of patient safety competence.

**Supplementary Information:**

The online version contains supplementary material available at 10.1186/s12912-024-01817-z.

## Introduction

Patient safety has always been an essential question in the medical field and it is also the core indicator to evaluate the quality of medical care [1]. As the largest medical professional group, clinical nurses, who closely engage in clinical nursing work and provide high-quality physical and psychological care for patients, directly provide patients with continuous health monitoring and nursing coordination services. They play an important role in ensuring the safety of patients through frequent, close and continuous contact with patients [2]. Therefore, it is particularly important to improve the clinical nurses’ attention to patient safety issues, improve the competency of nurses in dealing with complex medical safety issues, and avoid preventable nursing adverse events. PSC refers to the knowledge, attitude and skills that medical staff should possess to prevent patients from being injured by medical accidents [3]. The PSC of nurses has a direct impact on the patient’s safety and rehabilitation [4]. There has been convincing evidence that nurses are reliable information reporters for evaluating patient safety results (including adverse events) [5]. However, facing the risks of the medical field, the diversity of care types, and the complexity of technology, medical errors and nursing adverse events are frequent occurrences. According to the National Report on the Services, Quality and Safety in Medical Care System released in 2017–2018, a sample survey of 7855 hospitals nationwide pointed out that elementary operation of nursing and management errors rank second with 15.51%. Working experience [6], professional quality [7], working fatigue [8] and other factors all can cause safety problems for patients.

Now medical workers pay more attention to nurses’ PSC level and strive to explore methods to promote nurses’ competency in medical care. Related courses have been set up [9] and corresponding evaluation tools have been developed [10]. The evaluation contents involve knowledge, skills and attitudes, related to medical error reporting, safety culture, attitude towards medical errors and handling of uncertain events. These contents are widely used in maintaining patient safety, and are also essential competencies for medical personnel in team cooperation. In China, the framework of PSC has been deeply studied [11], and targeted assessment questionnaires have been developed [12, 13], which provide effective evaluation tools for accurately evaluating the PSC level of nursing staff, reflecting on educational defects, improving teaching content and methods. However, the measurement of PSC is mainly based on the score, ignoring the heterogeneity between different types of nurse groups. LPA is an individual-centered research path [14]. Based on the combination of explicit variable characteristics of different individuals, it identifies different categories of subgroups contained in the tested group [15]. Different from the variable-centered research that emphasizes homogeneity, it emphasizes the individual heterogeneity between samples, and takes the group type as a variable to accurately analyze the characteristics of different groups. Under the premise of the same PSC score, there are differences in the emphasis on the abilities of different nurses. Some nurses have stronger knowledge and skills, while others have better communication skills. Therefore, this study used LPA to explore the differences in the characteristic distribution of nurse PSC in different dimensions.

Furthermore, the articles on influencing factors of PSC are relatively few and mainly focus on demography, which is relatively single. Some studies have pointed out that PSC is not only related to nurses’ psychological cognition and behavior factors [16] but also affected by organizational context factors [17]. Error management climate is a variable at the group or organization level. It is also the common perception of employees on the organization’s practice and behavior which is related to error communication, error knowledge sharing, error environment improvement and error handling [18]. Error management climate is an important part of nursing safety management, an important element in the framework of patient safety competency, which could directly affect nurses’ work attitudes and behavior, and thus affect patient safety [12]. Psychological safety is a common belief among team members regarding the safety of interpersonal relationships. The improvement of psychological security could effectively enhance employees’ safety performance [19]. In the organizational environment of the hospital, psychological security is the key subjective factor affecting the patients’ safety. A higher sense of psychological security could encourage nurses to comply with the patient safety system and promote the active reporting of errors and accidents [20]. Proactive behavior focuses on self-initiated and future-oriented action that aims to change and improve the situation or oneself [21]. In clinical nursing situations, proactive behavior is shown as nurses actively taking measures to prevent, observe, report, deal with and feedback on patients’ safety problems, to ensure patients’ safety and improve their PSC.

Therefore, the research questions of this study were “what latent profiles exist of PSC among nurse groups and what factor can influence nurses’ PSC at different latent profiles?”. To address the above questions, two main processes were conducted in our study. Firstly, this article used LPA to identify the latent profiles of clinical nurses’ PSC and to compare the differences in PSC among different latent profiles. Secondly, combined with demographic factors, this paper introduced error management climate, psychological security and proactive behavior, to explore their impact on PSC in different latent profiles. The study’s purpose was to deeply explore the impact of organizational-level error management climate on individual-level PSC. At the same time, it comprehensively considered the nurses’ personal psychological cognition and behavioral factors, and analyzed the importance of psychological security and subjective behavior in the process of cultivating PSC. In formulating strategies to enhance nurses’ PSC, managers could comprehensively consider organizational environmental, individual psychological cognitive and behavioral factors, and attach importance to the error management climate, psychological security, and proactive behavior.

## Methods

### Design

The study was a cross-sectional survey and adhered to the STROBE guideline for cross-sectional studies.

### Participants

A total of 816 clinic nurses from a hospital in Harbin were recruited from June to August 2022. The samples came from a total of 52 departments across six major systems: internal medicine, surgery, gynecology, pediatrics, emergency and ICU. The inclusion criteria for this study were as follows: Registered nurses (RNs) with professional qualifications; Nurses with more than one year of work experience; Working in the clinical; Informed consent; Voluntary participation in this study. The exclusion criteria: The invalid questionnaire would be eliminated which had obvious logical errors, strong regularity of answers or too short answer time.

### Sample size

The sample size was calculated from 8 times the item under test [22]. There were 78 items in this questionnaire. Therefore, the calculation formula of sample size was *N* = (8 + 16 + 16 + 9 + 29) * 8 = 624, which means that at least 624 subjects were required for this study. At the same time, considering the sample loss rate of 20%, the sample size should be further expanded. Therefore, the minimum sample size required was *N* = 624*(1 + 20%) ≈ 749.

### Data collection

The questionnaires were sent to the nursing manager of each ward, who delegated the investigation task to each clinical department in an electronic format (https://www.wjx.cn/vm/tov7q3F.aspx). Investigation contents included purposes, filling methods and the main content. The participants were informed that the study was anonymous and voluntary, and the filling time was 15–30 min. The completed documents should be submitted immediately to ensure their validity and effectiveness. Data verification and entry should be completed by two persons. A total of 816 questionnaires were eventually completed, but those that had obvious logic errors (*n* = 6), strong regularity (*n* = 9), or answers with less than 3 min (*n* = 19) were excluded. Finally, 782 valid questionnaires were recovered, with an effective recovery rate of 95.8%.

### Instruments

#### Participant characteristics

The demographic characteristics of nurses were measured using an 8-item self-administered questionnaire. Demographic characteristics included sex, age, working years, professional title, educational level, marital status, employment form, and department.

#### Error management climate scale

Error management climate was measured using the error management climate scale. It was used to assess individual perceptions of the organization’s practices and behaviors regarding error handling. It was prepared by van Dyck et al. [18]. There are 16 items in total in 4 dimensions, including error learning (4 items), error thinking (5 items), error ability (3 items) and error communication (4 items). The Likert 5-level scoring method is adopted, and the score range is 16 ~ 80. The higher the score, the better of error management atmosphere perceived by nurses. The correlation coefficient between four factors and the total scale score is 0.856 and 0.950. This indicates that the scale has good content validity. The Cronbach’s α coefficient was 0.875.

#### Security questionnaire

Psychological safety was measured using a security questionnaire. It was compiled by Zhong et al. [23] and used to evaluate individual psychological characteristics. It includes two dimensions: the sense of control (8 items) and interpersonal security (8 items), with a total of 16 items. The Likert 5-level scoring method is adopted, and the total score ranged from 16 to 80. The higher the score, the stronger the sense of psychological security. The correlation coefficient between the two factors and the total scale score is 0.857 and 0.870. This indicates that the scale has good content validity. The Cronbach's α coefficient is 0.875.

#### Proactive behavior performance scale

Proactive behavior was measured using the proactive behavior performance scale. It was prepared by Griffin et al. [24] and used to evaluate the frequency of individual proactive behavior. There are 9 items in 3 dimensions, including individual (3 items), team (3 items) and organization (3 items). The scale mainly measures the frequency of proactive behaviors among nurses over the past month. The Likert 5-level scoring method is adopted, with a total score range of 9 ~ 45. The higher the score, the more active behaviors. The correlation coefficient between the three factors and the total scale score is 0.858 and 0.963. This indicates that the scale has good content validity. The Cronbach’s α coefficient is 0.920.

#### Patient safety competency self-rating scale of nurses

PSC was measured using the patient safety competency self-rating scale of nurses. It was developed by Wei et al. [13], it includes the knowledge factor (10 items), system factor (8 items), attitude factor (5 items) and skill factor (6 items), with a total of 29 items in four dimensions. Likert 5-level scoring method is used, with a total score range of 29–145. The higher the score, the stronger the nurse's PSC. Each factor is highly correlated with the total score, with a correlation coefficient of 0.619 ~ 0.822. This indicates that this scale had good content validity. The Cronbach's α coefficient was 0.930.

### Data analysis

To address the primary purpose of this study, we performed LPA to categorize the nurses into latent subgroups based on their responses on the four dimensions of the PSC scale. LPA was performed using Mplus version 8.3 statistical software. The analysis started from the single category model, and the number of categories in the model gradually increased until the fitting index reached the best. These indicators included: (1) log-likelihood ratio test (Log(L)), Akaike information criteria (AIC), Bayesian information criteria (BIC) and adjusted BIC (ABIC). Lower values indicate better fit [25]; (2) The entropy value is a statistic that measures the probabilistic accuracy of classification into a latent class, with values closer to 1.0 indicating high entropy, which is desirable. (3) Lo-Mendell‐Rubin (LMR) test and the Bootstrap Likelihood Ratio Test (BLRT) are used to measure how much the model improved with the addition of profiles based on changes in *p* values [26]. It was also necessary to determine the final number of categories in combination with the practical significance of classification. After the best fitting model was selected, multiple logistic regression analysis was used to explore the influencing factors of patient safety competency. Finally, Wald’s test was used to examine significant differences in indicators across profiles.

Once the optimal number of latent profiles had been identified, the nurses were classified into latent profile groups based on the most likely latent class membership. Data were analyzed using SPSS version 26.0 software. Firstly, we used univariate analyses, including one-way ANOVA and the chi-square test, to identify variables with statistically significant differences among the PSC subgroups. Variables that were significant in the univariate analysis (*p* < 0.05, two-sided probability) were entered into the multinomial logistic regression model. *p* < 0.05 was set as the threshold for the inclusion of a variable in the final model.

## Results

### Participant characteristics

Among the 782 nurses, 737 were female (94.2%). More than half of the nurses was aged 31 ~ 40 years (59.2%). The proportion of people who have worked for 5 ~ 10 years was the largest. More than half of nurses (52.7%) hold primary professional titles. The educational background is mainly undergraduate (89.3%). Internal medicine and surgery account for over two-thirds of nurses. Married individuals account for 66.1%. More than half of the nurses (76.6%) are employed through contracts. The detailed participant characteristics are listed in Table 1.

**Table 1:** Participants’ demographic characteristics (*n* = 782)

Variable	Number	Proportion (%)
Gender		
Male	45	5.75
Female	737	94.25
Age(year)		
< 25	14	1.79
25–30	180	23.02
31–40	463	59.21
41–50	99	12.66
> 50	26	3.32
Working years		
< 5	207	26.47
5–10	353	45.14
11–15	186	23.79
> 15	36	4.60
Professional titles		
Primary	412	52.69
Intermediate	338	43.22
Senior	32	4.09
Education		
Junior college	53	6.78
Undergraduate	698	89.26
Graduate	31	3.96
Marital status		
Married	517	66.11
Unmarried	206	26.34
Divorced/widowed	59	7.54
Employment form		
Contract	599	76.60
Staff	183	23.40
Departments		
Internal medicine	356	45.52
Surgery	227	29.02
Gynecology	52	6.65
Pediatrics	44	5.63
Emergency	57	7.29
ICU	46	5.88

### Descriptive statistics and correlations

Nurses’ PSC was positively related to error management climate, and psychological safety and proactive behavior. The PSC score was 121.31 (SD = 19.51). This result showed that the PSC of clinical nurses was at the level of the medium on the high side. The error management climate score was 70.28 (SD = 11.93), which was at a relatively high level. The psychological safety score was 61.21 (SD = 13.44), indicating a moderate to low level. The proactive behavior score was 37.60 (SD = 7.33), which was at a high level. The results were shown in Table 2.

**Table 2:** Descriptive statistics and correlations between PSC, error management climate, psychological safety and proactive behavior

	M	SD	1	2	3	4	5	6	7	8
1 PSC	121.31	19.51	1							
2 Knowledge factor	41.65	7.24	0.942^**^	1						
3 System factor	34.27	5.75	0.949^**^	0.843^**^	1					
4 Attitude factor	21.25	3.49	0.866^**^	0.723^**^	0.836^**^	1				
5 Skill factor	24.14	4.65	0.903^**^	0.805^**^	0.800^**^	0.720^**^	1			
6 Error management climate	70.28	11.93	0.584^**^	0.538^**^	0.572^**^	0.528^**^	0.507^**^	1		
7 Psychological safety	61.21	13.44	0.560^**^	0.526^**^	0.536^**^	0.455^**^	0.525^**^	0.366^**^	1	
8 Proactive behavior	37.60	7.33	0.645^**^	0.625^**^	0.611^**^	0.502^**^	0.596^**^	0.424^**^	0.556^**^	1

^**^Significant at the 0.01level

### Exploratory latent profile analysis

**Fig. 1:**
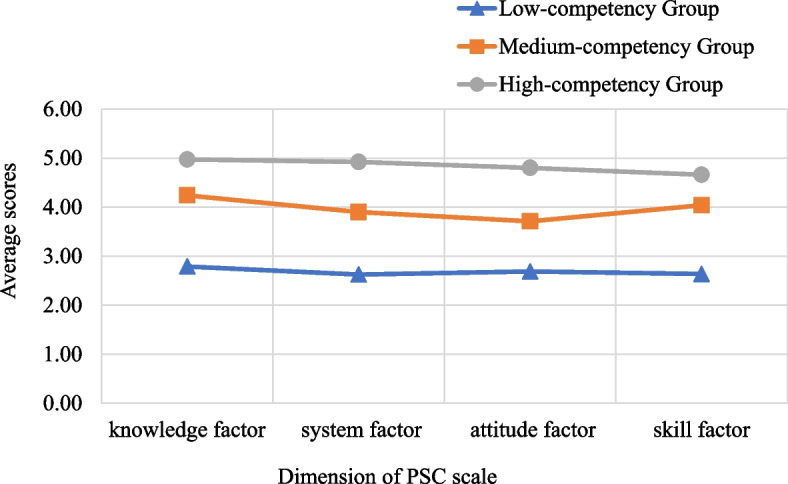
Different profiles of the PSC among clinical nurses

This study selected three profiles as the best-fitting model, which had the lower AIC (4461.255), BIC (4545.168), and aBIC (4488.009). The *P*-values of the LMR test (< 0.0319), and BLRT (< 0.001) were the smallest, suggesting that this model was statistically significant at the α = 0.05 level. The results were shown in Table 3; Fig. 1. As illustrated in Fig. 1, C1 profile and C3 profile had the lowest and highest scores in all dimensions, and were thus defined as the “Low-competency Group” and “High-competency Group”, accounting for 9.5% (74/782) and 42.2% (330/782), respectively. C2 profile, in which the scores were intermediate across the four dimensions, was labeled as the “Medium-competency Group”, accounting for 48.3% (378/782).

**Table 3:** Goodness‐of‐fit statistics for latent profile analyses

Number of classes	K	Log‐likelihood	AIC	BIC	aBIC	Entropy	Proportions	LMR	BLRT
1	8	-3447.942	6911.884	6949.178	6923.774	—	—	—	—
2	13	-2819.932	5665.864	5726.468	5685.186	0.863	0.542/0.458	0.0495	< 0.001
**3**	**18**	**-2212.628**	**4461.255**	**4545.168**	**4488.009**	**0.952**	**0.095/0.483/0.422**	**0.0319**	**< 0.001**
4	23	-1827.135	3700.270	3807.493	3734.456	0.976	0.469/0.014/0.426/0.091	0.0341	< 0.001
5	28	-1695.351	3446.703	3577.235	3488.321	0.977	0.014/0.384/0.092/0.128/0.382	0.3113	< 0.001

Bold values indicate the optimal model

*Abbreviations*: *K* Number of Free Parameters, *AIC* Akaike Information Criterion, *BIC* Bayesian Information Criterion, *aBIC* Adjusted BIC, *LMR* Lo‐Mendell‐Rubin Test, *BLRT* Bootstrap Likelihood Ratio Test, —Not applicable

### Characteristics of latent profile membership

Univariate analysis showed that age, working years, professional titles, education and departments were significantly different in the three latent profiles. The scores of all dimensions of nurses’ PSC in the Low-competency Group were significantly lower than the overall level. Nurses in the Low-competency Group accounted for a larger proportion in terms of working years < 5 years, primary professional titles. The proportion of nurses in the Medium-competency Group was the largest and the nurses in this group were mainly composed of 31–40 year, undergraduate, and had worked for 5–10 years. The score of each dimension in the High-competency Group was the highest. The composition of nurses in this group was dominated by intermediate and senior professional titles, and the number of working years 11–15 years was the most. Furthermore, compared to the Low and Medium subgroups, the High-competency Group had significantly higher scores in error management climate, psychological security and proactive behavior. Detailed information can be found in Table 4.

**Table 4:** The differences in nurses’ PSC latent profiles in demography, error management climate, psychological safety and proactive behavior (*n* = 782)

Variable	Respondents	Low-competency	Medium-competency	High-competence	χ^2^/F	*p*
Gender						
Male	45 (5.75%)	6 (8.11%)	18 (4.76%)	21 (6.36%)	2.799	0.247
Female	737(94.25%)	68 (91.89%)	360 (95.24%)	309 (93.64%)		
Age(year)						
< 25	14 (1.79%)	1 (1.35%)	8 (2.12%)	5 (1.52%)	31.489	< 0.001^*^
25–30	180 (23.02%)	18 (24.32%)	93 (24.60%)	69 (20.91%)		
31–40	463 (59.21%)	50 (67.56%)	216 (57.14%)	197 (59.70%)		
41–50	99 (12.66%)	4 (5.41%)	42 (11.11%)	53 (16.06%)		
> 50	26 (3.32%)	1 (1.35%)	19 (5.03%)	6 (1.82%)		
Working years						
< 5	207 (26.47%)	38 (51.35%)	85 (22.49%)	84 (25.45%)	86.806	< 0.001^*^
5–10	353 (45.14%)	5 (6.76%)	206 (54.50%)	142 (43.03%)		
11–15	186 (23.79%)	23 (31.08%)	79 (20.90%)	84 (25.45%)		
> 15	36 (4.60%)	8 (10.81%)	8 (2.12%)	20 (6.06%)		
Professional titles						
Primary	412 (52.69%)	70 (94.59%)	191 (50.53%)	151 (45.76%)	76.277	< 0.001^*^
Intermediate	338 (43.22%)	2 (2.70%)	177 (46.83%)	159 (48.18%)		
Senior	32 (4.09%)	2 (2.70%)	10 (2.65%)	20 (6.06%)		
Education						
Junior college	53 (6.78%)	11 (14.86%)	22 (5.82%)	20 (6.06%)	26.859	< 0.001^*^
Undergraduate	698 (89.26%)	62 (83.78%)	347 (91.80%)	289 (87.58%)		
Graduate	31 (3.96%)	1 (1.35%)	9 (2.38%)	21 (6.36%)		
Marital status						
Married	517 (66.11%)	52 (70.27%)	245 (64.81%)	220 (66.67%)	2.610	0.625
Unmarried	206 (26.34%)	19 (25.68%)	101 (26.72%)	86 (26.06%)		
Divorced/widowed	59 (7.54%)	3 (4.05%)	32 (8.47%)	24 (7.27%)		
Employment form						
Contract	599 (76.60%)	53 (71.62%)	290 (76.72%)	256 (77.58%)	1.426	0.490
Staff	183 (23.40%)	21 (28.38%)	88 (23.28%)	74 (22.42%)		
Departments						
Internal medicine	356 (45.52%)	38 (51.35%)	185 (48.94%)	133 (40.30%)	107.062	< 0.001^*^
Surgery	227 (29.02%)	17 (23.97%)	127 (33.60%)	83 (25.15%)		
Gynecology	52 (6.65%)	7 (9.46%)	20 (5.29%)	25 (7.58%)		
Pediatrics	44 (5.63%)	7 (9.46%)	22 (5.82%)	15 (4.55%)		
Emergency	57 (7.29%)	1 (1.35)	16 (4.23%)	40 (12.12%)		
ICU	46 (5.88%)	4 (5.41%)	8 (2.12%)	34 (10.30%)		
Error management climate	70.28 ± 11.93	55.53 ± 17.42	68.04 ± 8.07	76.15 ± 10.32	139.768	< 0.001^*^
Psychological safety	61.21 ± 13.44	47.18 ± 13.28	57.56 ± 10.02	68.54 ± 12.70	140.120	< 0.001^*^
Proactive behavior	37.60 ± 7.33	27.68 ± 8.43	35.97 ± 5.99	41.69 ± 5.36	191.109	< 0.001^*^

^*^Significant at the 0.05 level

### Predictors of latent profile membership

We then performed multinomial logistic regression to validate the influencing factors of PSC in three latent profiles, using the High-competency Group as the reference. As shown in Table 5. Compared with the High subgroup, the Low-competency Group nurses were more likely to have intermediate professional titles, and gynecology and emergency departments were more likely to be grouped into the Low group. The Medium-competency Group nurses were more likely to work less than 10 years. There was a high possibility that internal medicine, surgery and pediatrics departments would be assigned to the Medium subgroup. Both groups had a lower level of error management climate, psychological security and proactive behavior.

**Table 5:** The multifactor analysis of nurses’ PSC by logistic regression (*n* = 782)

	High-competency(refer)^a^vs						Medium-competency					
	Low-competency											
	B	SE	Wald χ^2^	p	OR	95% CI	B	SE	Wald χ^2^	p	OR	95% CI
Constants	21.344	3.303	41.749	< 0.001^*^	—	—	11.280	1.542	53.517	< 0.001^*^	—	—
**Ages**												
< 25	0.425	2.044	0.043	0.835	1.529	(0.028, 84.084)	-0.488	0.956	0.260	0.610	0.614	(0.094, 4.002)
25–30	0.657	1.638	0.161	0.688	1.929	(0.078, 47.845)	-0.690	0.634	1.186	0.276	0.502	(0.145, 1.736)
31–40	1.489	1.564	0.906	0.341	4.432	(0.207, 95.105)	-0.747	0.568	1.733	0.188	0.474	(0.156, 1.441)
41–50	2.714	1.786	2.309	0.129	15.089	(0.455, 499.967)	-0.856	0.597	2.056	0.152	0.425	(0.132, 1.369)
> 50(refer)												
**Working years**												
< 5	1.291	0.859	2.260	0.133	3.636	(0.676, 19.574)	1.759	0.570	9.512	0.002^*^	5.808	(1.899, 17.766)
5–10	-1.619	0.946	2.931	0.087	0.198	(0.031, 1.264)	1.520	0.540	7.929	0.005^*^	4.574	(1.587, 13.181)
11–15	0.178	0.854	0.043	0.835	1.195	(0.224, 6.373)	0.998	0.565	3.119	0.077	2.713	(0.896, 8.216)
> 15(refer)												
**Education**												
Junior college	1.617	2.092	0.598	0.439	5.038	(0.084, 303.885)	0.219	0.681	0.103	0.748	1.244	(0.327, 4.732)
Undergraduate	1.965	1.981	0.984	0.321	7.138	(0.147, 346.607)	0.758	0.536	1.999	0.157	2.134	(0.746, 6.101)
Graduate (refer)												
**Professional titles**												
Primary	-0.573	1.500	0.146	0.702	0.564	(0.030, 10.656)	0.321	0.586	0.301	0.583	1.379	(0.438, 4.345)
Intermediate	-3.936	1.582	6.190	0.013^*^	0.020	(0.001, 0.434)	0.285	0.540	0.279	0.598	1.330	(0.461, 3.832)
Senior (refer)												
**Departments**												
Internal medicine	-1.330	0.864	2.371	0.124	0.264	(0.049, 1.438)	1.060	0.514	4.251	0.039*	2.888	(1.054, 7.913)
Surgery	-1.483	0.925	2.574	0.109	0.227	(0.037, 1.389)	1.314	0.526	6.232	0.013*	3.720	(1.326, 10.434)
Gynecology	-2.519	1.152	4.780	0.029^*^	0.081	(0.008, 0.770)	0.362	0.622	0.339	0.560	1.436	(0.425, 4.856)
Pediatrics	-0.129	1.324	0.009	0.922	0.879	(0.066, 11.784)	1.946	0.626	9.671	0.002*	6.998	(2.053, 23.849)
Emergency	-4.058	1.524	7.087	0.008^*^	0.017	(0.001, 0.343)	0.084	0.620	0.018	0.892	1.088	(0.323, 3.666)
ICU(refer)												
**Error management climate**	-0.152	0.019	62.317	< 0.001^*^	0.859	(0.827, 0.892)	-0.083	0.013	41.987	< 0.001^*^	0.921	(0.898, 0.944)
**Psychological safety**	-0.095	0.019	25.405	< 0.001^*^	0.909	(0.876, 0.943)	-0.051	0.009	29.834	< 0.001^*^	0.950	(0.933, 0.968)
**Proactive behavior**	-0.213	0.032	43.976	< 0.001^*^	0.808	(0.759, 0.861)	-0.118	0.019	37.231	< 0.001^*^	0.889	(0.856, 0.923)

*Rrefer* Reference group

^*^Significant at the 0.05 level

^a^High-competency Group profile as the reference category

## Discussions

### Latent profile of clinical nurses’ PSC and application in nursing practice

Through latent profile analysis, this study found that clinical nurses’ PSC could be divided into three profiles: Low-competency Group, Medium-competency Group and High-competency Group. It was suggested that there were significant individual differences in the level of nurses’ PSC. The Medium-competency Group had the largest number (48.3%), which accounted for about half of the total, followed by the High-competency Group (42.2%), and the Low-competency Group had the least number (9.5%). This result showed that the PSC of clinical nurses was at the level of the medium on the high side, which was consistent with the research results of Zhao et al. [27].

The scores of all dimensions of nurses’ PSC in the Low-competency Group were significantly lower than the overall level. As a possible explanation for this, nurses in this group needed to be strengthened in patient safety knowledge and skills, and lacked understanding of relevant systems and processes of patient safety management systems and events. It could be known from Table 4 that nurses in the Low-competency Group accounted for a larger proportion in terms of working years < 5 years, and primary professional titles. It showed that the nurses in this group were affected by their working time and educational background. The primary title nurses had a short working time, and their clinical experience needed to be gradually accumulated. Meanwhile, the reserves of professional knowledge and skills related to patient safety were insufficient, their ability to assess and judge patient safety risks was short, and the nursing risk was higher [28]. Our findings could prompt managers to strengthen the training of patients’ safety-related knowledge and skills of Low-competency Group nurses. And the sharing and reporting behaviors should be encouraged [29], to improve the level of PSC and ensure the safety of patients.

The Medium-competency Group was currently the main practitioner of clinical nursing work, with rich clinical practice experience and highly patient safety-related knowledge and skills. They had better qualifications and their PSC level was high. It was noteworthy that compared with the scores of nurses in the Medium-competency Group in the four dimensions, the score of the attitude factor was the lowest (Fig. 1). The finding showed that the awareness of patient safety needed to be improved, and there might be a mentality of fearing punishment and reluctance to share and report with colleagues when dealing with patient safety-related adverse events. Nursing managers should focus on correcting the cognitive attitude of nurses toward patients’ safety problems, build and apply an effective, non-punitive adverse event reporting system and give immediate feedback on events [30]. Further, managers would encourage nurses to share the handling methods and experience of patients’ safety-related events, guide them to establish a correct cognitive attitude, and improve their PSC level.

The score of each dimension in the High-competency Group was the highest. The composition of nurses in this group was dominated by middle and senior professional titles, and the number of working years > 15 years was the highest. As the improvement of PSC was a gradual process, the long-term accumulation of experience would help nurses better identify the risk of adverse events and reduce the incidence of errors [27]. At the same time, the promotion of professional titles required sufficient clinical working years and a solid foundation of professional knowledge and skills, which reflected the technical level and working ability of nurses to a certain extent. Therefore, when nurses faced and dealt with various problems related to patient safety, the longer and higher working age and professional titles they had, the more comprehensive would they be in nursing safety knowledge and operation skills. Their richer clinical experience meant that they had a sharper insight into existing or potential safety problems [31]. Managers should give full play to the characteristic advantages of the nurses in the High-competency Group, encourage employees to share more nursing points and experiences, and create a good patient safety culture atmosphere to improve the overall nurses’ PSC.

### The influence of demographic characteristics on nurses’ PSC

Our study found that working years, professional titles and departments were the influencing factors of nurses’ PSC by univariate and multinomial logistic regression analyses. It was consistent with the research results of Zhao et al. [27].

Working years are closely related to clinical experience and professional title promotion. The nurses with long-term clinical practice accumulation have rich experience in contacting and dealing with patients’ safety-related problems. And they also have a stronger ability to evaluate and predict patients' safety situations, and could find and solve potential safety hazards that would lead to adverse consequences as soon as possible [32].

The promotion of nurse professional titles requires sufficient clinical working years and passing the examination of professional-related knowledge and skills. Nurses who have higher professional titles would experience longer working time and had already reserved strong professional knowledge and skills. Therefore, nurses with high seniority and professional titles would have superior PSC levels. The results showed that there was no significant difference between Medium and High in the level of professional titles. The possible explanation was that the professional titles in the two groups were mainly primary and intermediate, and the number of people was relatively balanced, which couldn’t highlight the difference.

Departments were the influencing factor of PSC in this article. Our study results showed that the PSC level of medical and surgical nurses in the Medium-competency Group was higher than that of emergency and ICU nurses. Consistent with the research of Zhao et al. [27]. Most emergency patients are in critical condition, and nurses need to bear high-intensity and high-load working conditions for a long time, which is prone to safety accidents [33]. However, the result was inconsistent with Zhao et al. [34]. Zhao believed that ICU nurses mainly cared for acute and critical diseases. They had a stronger sense of safety and were better at observing and analyzing patients’ conditions. The PSC level would be higher than other departments. It was a pity that the number of emergency and ICU samples collected in this study was small, which would affect the interpretation of the results.

The multinomial Logistic regression analysis found that education was not the influencing factor of PSC, consistent with the research of Abdul et al. [35]. Although the nursing educational level was not associated with the outcomes of care, training programs could increase their self-confidence, knowledge, critical thinking ability and improve their interpersonal skills. However, this study result was inconsistent with Kerfoot et al. [36]. Kerfoot’s research showed that nurses with a master's degree or higher would have more opportunities to participate in patient safety-related theme learning projects, which could help improve nurses’ confidence in patient safety practice. However, the proportion of the number with a master’s degree or above in the total number was only 4.0% (31/782), and the three groups of nurses were mainly with bachelor’s degrees, which weakened the ability difference that would be caused by the graduate degree.

According to the above results, the improvement of clinical nurses’ PSC is a long-term, continuous and gradual process. Managers could formulate learning and training on patients’ safety by stages and professional titles. For key departments, such as emergency, managers should expand the implementation power to improve patients' safety awareness and improve the nurses’ PSC level.

### The influence of error management climate, psychological security and proactive behavior on nurses’ PSC

Formerly, nursing errors have been treated with a strict cognitive attitude, and erroneous behaviors have been dealt with through dignified or punishment. However, the error is inevitable. What is more important must be to learn from experience and avoid risks after mistakes occur. Errors should be treated with a more scientific attitude. Some studies had shown that scientific error management could create a positive error management climate [37]. Managers should pay attention to error management and error prevention, and guide nurses to have a positive understanding of errors. Nurses could turn the self-blame thinking after errors into learning-oriented thinking, weaken the frustration and guilt of nurses due to errors, and actively use their own and surrounding available resources to solve the negative impact of errors. A positive error management climate could encourage nurses to take the initiative in reporting errors and accidents, and promote the discussion and learning of errors and accidents [38]. Furthermore, it also could make nurses pay more attention to maintaining patient safety and stimulate their PSC levels.

The psychological security of nurses includes the sense of interpersonal security and the sense of control, which are affected by the interpersonal relationships of surrounding organizations and factors of control over work. In free and healthy interpersonal communication, nurses would trust and rely on the hospital organization from the bottom of their hearts, and then take the initiative to establish a trusting relationship with others. Good personal relationships could encourage nurses to actively explore their potential, and stimulate more constructive behaviors conducive to the safety of patients [39]. At the same time, nurses with a high sense of work control have stronger dominance over the working environment, working methods and work quality, which is conducive to increasing the investment in work [40]. They have a stronger sensitivity and professionalism to discover and solve patient safety problems, and their PSC levels are higher.

The generation of nurses’ proactive behavior needs to rely on the support of a strong self-attainment and organizational environment [41]. Psychological security and scientific error management are important guarantees for the generation of active behavior. In a positive organizational error management climate, nurses’ error behaviors are correctly evaluated and treated, and also their psychological security is satisfied. They could obtain knowledge and experience from errors to improve the internal driving force and reduce the possible risks or losses caused by active behaviors. Furthermore, reasonable error solutions could stimulate nurses’ positive emotions and intrinsic motivation, and encourage more proactive behaviors, which have great significance for nurses to improve their PSC [42].

### Limitations and prospects

Firstly, the article was a cross-sectional survey, which was limited in description and exploration. Due to the epidemic situation during the research period. Since the study was carried out during the COVID-19 epidemic, the objects only came from one hospital, which would have information bias. In the future, the longitudinal research design should be used to carry out multi-center and large-sample surveys to further verify and improve the conclusions of this study. Secondly, the study did not investigate nurses’ situation with patient safety education courses, training or learning. And there was a lack of exploration of the impact of patient safety education on competency. The results should be supplemented in the next to provide a reference for managers to formulate targeted training programs. Thirdly, the research tools were in the form of self-reporting, which could not avoid the subjectivity of the research object. In the future, more objective tools should be used to measure nurses’ PSC at a future date.

## Conclusions

This study used LPA to evaluate clinical nurses’ PSC levels. The results showed that there were obvious classification characteristics in clinical nurses’ PSC, which were divided into three latent profiles: Low-competency Group, Medium-competency Group and High-competency Group. The study results also showed that the working years, professional titles, departments, positive error management climate, psychological security and proactive behavior were the influencing factors on PSC. This study will help improve nurses’ PSC, which is currently an important issue. The priority for promoting PSC is to improve nurses’ practical ability by acquiring knowledge and skills through education and training. Also, managers should pay attention to the continuous training of clinical nurses’ PSC, and implement targeted interventions for nurses in different work stages, professional titles and departments. Furthermore, they could use efficient management strategies to create a positive error management climate, give nurses more psychological security and stimulate more proactive behaviors in patient safety management, which could constantly improve the PSC levels.

### Supplementary Information

**Supplementary Material 1. **

## Data Availability

The data supporting the findings of this study are available on request from the corresponding author. The data are not publicly available due to privacy or ethical restrictions.
